# Suppression of *Aspergillus fumigatus* Germination by Neutrophils Is Enhanced by Endothelial-Derived CSF3 Production

**DOI:** 10.3389/fmicb.2022.837776

**Published:** 2022-04-29

**Authors:** Wenxin Zhang, Dan He, Yunyun Wei, Shumi Shang, Dong Li, Li Wang

**Affiliations:** ^1^Key Laboratory of Zoonosis Research, Ministry of Education, Department of Pathogenobiology, Jilin University Mycology Research Center, College of Basic Medical Sciences, Jilin University, Changchun, China; ^2^Department of Immunology, College of Basic Medical Sciences, Jilin University, Changchun, China

**Keywords:** *Aspergillus fumigatus*, neutrophil, CSF3, germination, conidia

## Abstract

Infection with *Aspergillus fumigatus* can cause life-threatening diseases in immunocompromised patients with an unacceptable mortality rate. Angioinvasion is one of the features of severe invasive aspergillosis. Neutrophils are short-lived immune cells regulated by colony-stimulating factor 3 (CSF3) that play a key role in anti-fungal immune responses. To investigate the interactions between *A. fumigatus* and the host immune cells, such as neutrophils, we stimulated human umbilical vein endothelial cells (HUVECs) with the conidia of *A. fumigatus*, and co-cultured them with human neutrophils. Apoptosis and functions of neutrophils were analyzed. Our results showed that HUVECs upregulate the expression of CSF3, which could reduce the apoptosis of neutrophils while enhancing their functions. Lack of CSF3 was associated with enhanced apoptosis of neutrophils with impaired function. This work indicated that the CSF3 is required for neutrophil survival and function, at least in the early stages of *A. fumigatus* infection.

## Introduction

Aspergillosis remains a major health threat despite decades of studies and the availability of a range of anti-fungal treatments (Bandres et al., 2021). The mortality rate among immunocompromised patients, such as stem organ transplant recipients, patients with inherited immunodeficiency or acquired immune deficiency syndrome, can exceed 50% (Beer et al., 2018). *Aspergillus fumigatus* is the most common cause of aspergillosis and can be found in soil, air, and carbon-rich substrates. People usually inhale hundreds of conidia of *A. fumigatus* each day, but only in immunocompromised patients can it cause diseases, indicating that the immune system could recognize inhaled mold and avoid germination. Detailed knowledge about the interaction between the *A. fumigatus* and immune system could help develop a new treatment strategy in the treatment strategies for aspergillosis.

After the spore form of *A. fumigatus* is inhaled, the mucociliary clearance can clearly the inhaled conidia in healthy subjects. Recent studies have shown that alveolar macrophage and epithelial cells are important during the early stage of the *A. fumigatus* infections as dysfunctions of these cells lead to lung inflammation (Speirs et al., 2012; Burgel et al., 2016) or even angioinvasion if *A. fumigatus* causes invasive aspergillosis in lung and other organs (Kamai et al., 2006). If the *A. fumigatus* conidia colonize the lower airway, neutrophils are recruited to the fungal infection site as they are among the first line of defense against pathogens. In addition, unlike the macrophages, which usually attack conidia, neutrophils are the primary attackers on hyphae while phagocytizing conidia directly. Studies have also shown that neutrophils, but not macrophages, play an essential role in *A. fumigatus* infections (Mircescu et al., 2009). The anti-fungal substances, such as neutrophil extracellular traps (NETs) released by neutrophils, are required to control the conidia and germlings of *A. fumigatus* to prevent the invasive diseases (Gazendam et al., 2016).

Neutrophils are the most abundant cells among all leukocytes in the blood stream, and previous studies have shown that neutrophils play a central role in the immune response against infections. Not only can neutrophils kill pathogens directly *via* phagocytosis, releasing antimicrobial peptides, reactive oxygen species (ROS), and NETs but also they can initiate indirect killing *via* production of cytokines and chemokines to recruit and activate other immune cells to start a “full size” immune response. However, over-active or long-lasting activating neutrophils can cause damage to host tissues or even cause autoimmune diseases (Lehman and Segal, 2020). So, a well-organized “size” and “time” of neutrophil activation is critical to eradicating invading pathogens while avoiding detrimental side effects.

Multiple cytokines/chemokines play important roles in the recruitment and activation of neutrophils. The key protein that regulates neutrophil development, proliferation, differentiation, survival, and function is the granulocyte colony-stimulating factor (G-CSF), which is encoded by colony-stimulating factor (CSF) 3 gene (Panopoulos and Watowich, 2008). Although the protective role of G-CSF during infection-related diseases is well documented (Stroncek and Leitman, 1998; Hansakon et al., 2020), the role of G-CSF in *A. fumigatus* infections remains controversial (Lauruschkat et al., 2018; Martin et al., 2021). Therefore, a better understanding of the role of *CSF3* during *A. fumigatus* infections could provide more insight into the pathogenesis of invasive diseases caused by *A. fumigatus*.

Hence, these issues need to be investigated. We stimulated human umbilical vein endothelial cells (HUVECs) and human neutrophils with conidia of *A. fumigatus*, and analyzed neutrophils’ migration, survival, and functions upon stimulation. This study provides additional insight into the interaction between neutrophils/stromal cells and *A. fumigatus*.

## Materials and Methods

### Patients and Patient Samples

The study was approved by the Ethics Committee on Human Research of the First Hospital of Jilin University (approval No. 2018-250). Lung tissue samples were collected from the archives of the Department of Pathology at the First Hospital of Jilin University. There were 110 cases of lung tissue that had been surgically removed and were diagnosed without metastatic tumor between July 1, 2019 and July 31, 2020 at the First Hospital of Jilin University. Among them, 13 subjects were diagnosed with mold infection in the lung *via* periodic acid-Schiff (PAS) staining. The blood test results and basic characteristics of these 13 subjects were collected from the archives, and no personal data were collected (Table 1). Hematoxylin and eosin (H&E) stain pictures and unstained slides were kindly provided by the Department of Pathology, the First Hospital of Jilin University.

**TABLE 1:** Basic characters of subjects with mold infections.

Sex	Age	Diagnosis
F	48	Bronchiectasis
F	64	Bronchiectasis
F	62	Bronchiectasis
M	38	Bronchiectasis
F	53	Bronchiectasis
M	70	Bronchiectasis
M	46	Chronic obstructive pulmonary disease
F	41	Lung abscess
F	36	Pneumonitis
F	20	Pulmonary cysts
M	62	Pulmonary fibrosis
M	56	Pulmonary mycosis
F	30	Pulmonary sequestration

## Immunostaining

Human neutrophil elastase (ELA2) antibody (MAB91672, R&D Systems, MI, United States) was used to stain lung tissue sections, while horseradish peroxidase (HRP) congregated goat anti-rabbit IgG antibody (bs-0295G-HRP, Beijing Biosynthesis Biotechnology Co., Ltd., China) was used for immunohistochemistry (IHC) staining, and Cy3-labeled goat anti-mouse IgG antibody (6900-250, Biovision, CA, United States) was used for immunofluorescence staining. Images were taken using an Olympus CKX53 inverted microscope or an Olympus FV3000 confocal laser scanning microscope. Hematoxylin was used for IHC staining as a counter-stain. Detailed assay procedures were performed as previously described (Xu et al., 2019, 2021).

### Staining for *Aspergillus fumigatus*

Methenamine silver staining (MST, BA4094, BASO diagnostics, Inc., China) and Calcofluor white staining (CWS, 4404-43-7, Shanghai Maokang Biotechnology Co., Ltd., China) were used to label *A. fumigatus*. Both procedures were performed according to the protocol of the manufacturer.

### *Aspergillus fumigatus* Culture

*Aspergillus fumigatus* strain IFM40808 was used in this study. This strain was a gift from the Medical Mycology Research Centre of Japan Chiba University, which was isolated from the lungs of a 54-year-old female Japanese patient with invasive aspergillosis and then saved in the laboratory (Zhang et al., 2020). Conidia were inoculated on potato dextrose agar (PDA) medium (Becton Dickinson Co., Sparks, MD, United States) and grown for 4–6 days at 37°C. The fresh conidia were harvested in saline (0.9% NaCl, 0.01% Tween 20) isolated from mycelia using a cell strainer (40 μm), and counted using a hemocytometer.

### Cell Culture

Wild-type (WT) and *CSF3* knockout HUVECs were used in this study. All cells were grown in Dulbecco’s modified Eagle’s medium (DMEM, Sigma, MO, United States) supplemented with 10% heat-inactivated fetal bovine serum (FBS, BI, CT, US). WT HUVECs were stored in our laboratory (Tian et al., 2019). *CSF3* knockout HUVEC cells were generated using the CRISPR/Cas9 system, and the sequence for guide RNA (gRNA) used was CCG ACT TTG CCA CCA CCA TC, with the control gRNA CCG GGT CTT CGA GAA GAC CT. *CSF3* knockdown was achieved by transfection of siRNA against *CSF3* and control siRNA purchased from Guangzhou Ribo Biotechnology Co., Ltd. (stB0003446B, siN0000001, China). *CSF3* overexpressing was achieved by transfection of *CSF3* plasmid purchased from Sangon Biotech (Shanghai) Co., Ltd. (China). All transfection experiments were performed using Lipofectamine 2000 Transfection Reagent (11668019, Thermo Fisher Scientific, MA, United States) according to the instructions of the manufacturer.

### Conidia Germination Assay

HUVECs were seeded in a 24-well plate (3 × 10^5^ cells per well) for an overnight culture under normal tissue culture conditions. Fresh conidia of *A. fumigatus* were added to the cultured HUVEC cells in a 24-well plate as indicated in the manuscript. As described previously, light optical microscopy took photographs of spores at different time points (Wang et al., 2016).

### RNA Sequencing

HUVECs were seeded in a six-well plate (2 × 10^6^ cells per well) overnight before the conidia of *A. fumigatus* were added to the wells at a cell:spore ratio of 1:2. The cell lysates were harvested in Trizol (15596026, Thermo Fisher Scientific, MA, United States) 2 and 6 h post-stimulation and shipped to CapitalBio Technology in Beijing in dry ice for RNA sequencing and analysis. Three biological replicates were used for each condition.

### Neutrophil Isolation

Fresh samples of peripheral blood from healthy volunteers were collected in EDTA tubes (0102 EDTAK2, Kang Jian Medical, China) by venous puncture according to the approved protocol by the Ethics Committee on Human Research of the First Hospital of Jilin University (approval No. 2018-250). Blood was utilized within 4 h after the blood draw. Neutrophils were isolated using the EasySep Direct Human Neutrophil Isolation Kit according to the protocol of the manufacturer (19666, STEMCELL Technologies, BC, Canada).

### Neutrophil Migration and Co-culture in Transwell System

Isolated neutrophils suspended in DMEM containing 10% FBS at 10^6^/ml, 250 μl of cells were placed in the upper compartment of a Transwell chamber featuring an uncoated polyester membrane with 3- or 0.4-μm pores (3,472 and 3,470, Corning, NY, United States) after stimulating HUVECs with spores for 2 h in the bottom chamber. After incubation at the indicated time at 37°C and 5% CO_2_, the cells in the upper chamber of a 0.4-μm Transwell plate were harvested for quantitative polymerase chain reaction (qPCR) testing or flowcytometry. The supernatant was also harvested for the myeloperoxidase (MPO) assay. The suspended cells in the lower chamber of the 3-μm Transwell plate were counted using a hemocytometer.

### Western Blotting Analysis

Western blotting was performed as previously described (Xu et al., 2021). HUVECs were lysed in radioimmunoprecipitation assay (RIPA) buffer to detect G-CSF levels. Antibodies against G-CSF were obtained from Abcam (ab181053, United Kingdom), β-actin was obtained from Beijing Ray Antibody Biotech (RM2001, China), and goat anti-rabbit IgG/HRP and goat anti-mouse IgG/HRP were obtained from Beijing Biosynthesis Biotechnology (bs-0295G-HRP, bs-0296G-HRP, China).

### Polymerase Chain Reaction

RNA was purified from cells using TRIzol Reagent according to the instructions of the manufacturer (15596026, Thermo Fisher Scientific, MA, United States). Reverse transcription (RT) of RNA into cDNA was performed using a cDNA synthesis kit (Takara, Japan). Real-time PCR was performed using FastStart Universal SYBR Green Master (4913914001, Roche, Switzerland) on a Stratagene Mx3000P system (Agilent, CA, United States) (Tian et al., 2019). The primers used were as follows:

*CSF3* Forward 5′-AAG CTG GTG AGT GAG TGT GC-3′
Reverse 5′-GGC CAT TCC CAG TTC CA-3′
*IL1B* Forward 5′-GGG CCT CAA GGA AAA GAA TC-3′
Reverse 5′-TTC TGC TTG AGA GGT GCT GA-3′
*IL8* Forward 5′-GTG CAG TTT TGC CAA GGA GT-3′
Reverse 5′-CTC TGC ACC CAG TTT TCC TT-3′
*NOS2* Forward 5′-AGG TCC AAA TCT TGC CTG GG-3′
Reverse 5′-ATC TGG AGG GGT AGG CTT GT-3′
*TBP*  Forward 5′-ACA ACA GCC TGC CAC CTT AC-3′
Reverse, 5′-CTG AAT AGG CTG TGG GGT CA GG-3′

### Flow Cytometry

Neutrophils cultured in the upper chamber with 0.4-μm pores were harvested and then stained with an apoptosis analysis kit (AO2001-02A-G, Tianjin Sungene Biotech Co., China) for flow cytometry analysis on Guava easyCyte HT System (Luminex Corporation, TX, United States).

### Determination of Myeloperoxidase Activity

The supernatants from neutrophils cultured in the upper chamber with 0.4-μm pores and neutrophils cultured without Transwell inserts were harvested for myeloperoxidase (MPO) assay as previously described (Li et al., 2014; Xi et al., 2020).

### Statistical Analysis

Data are expressed as the means ± standard error of the mean (SEM). The statistical significance between the two groups was analyzed by Student’s *t*-test; one-way ANOVA followed by Tukey’s multiple *t*-tests were used when more than two groups were compared (Prism, GraphPad Software, CA, United States). The asterisk (*) represents *p* < 0.05 and was considered statistically significant. All experiments were repeated at least three times.

## Results

### Increased Recruitment of Neutrophils in *Aspergillus fumigatus* Infection Sites

**FIGURE 1:**
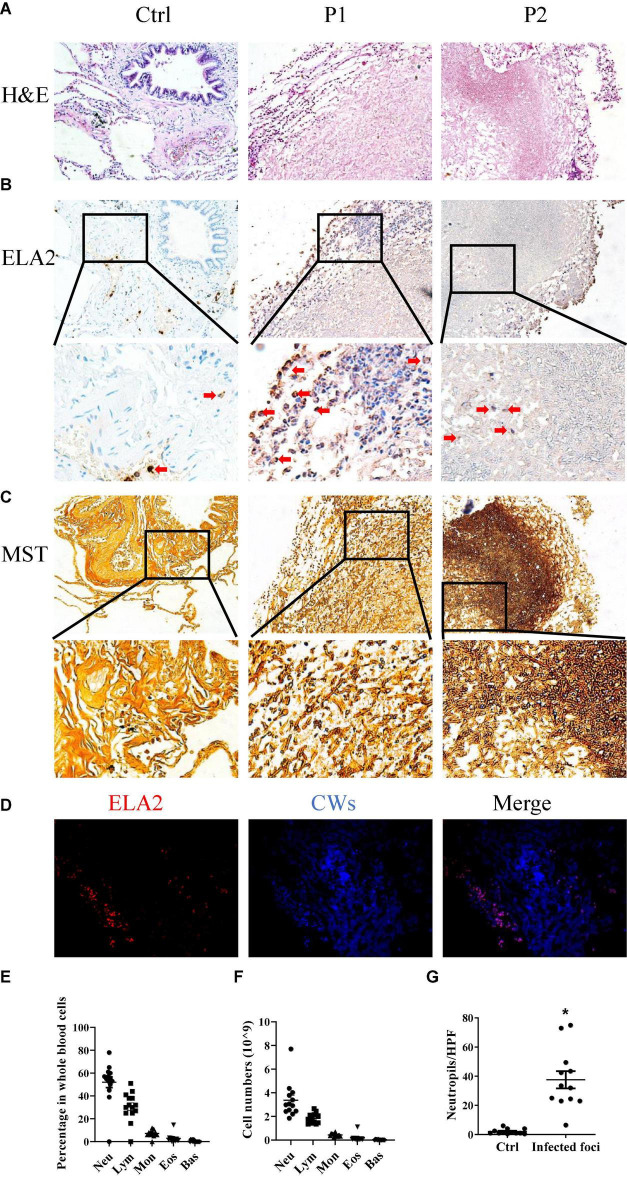
Neutrophils are involved in *Aspergillus* (*A). fumigatus* infection. Lung tissues obtained from patients with mold infection in the lungs were stained with **(A)** hematoxylin and eosin (H&E) staining, **(B)** immunohistochemical staining for human neutrophil elastase (ELA2), and **(C)** methenamine silver staining (MST). **(D)** Immunofluorescence staining for ELA2 and Calcofluor white staining (CWS) in lung tissue samples with *Aspergillus* spp. infections. Leukocyte percentages **(E)** and numbers **(F)** in the peripheral blood of patients with *Aspergillus* spp. infections. **(G)** Neutrophil numbers in the lung tissue sections were counted. Ctrl, lung tissue sections free from *Aspergillus* spp. Infection; P1 and P2, lung tissue sections with *Aspergillus* spp. infections. HPF, high power field. *p < 0.05. Original magnification × 100. The red arrow indicates neutrophils.

In order to investigate the interaction between the *A. fumigatus* and the host immune system, we selected 13 tissue samples from the archives of the Department of Pathology, the First Hospital of Jilin University, all of which were cleared from metastatic tumors (Table 1). Morphology analysis showed that there were neutrophils surrounding and infiltrating the fungal infection loci, while in healthy controls, there were very few neutrophils in the lung interstitium (Figures 1A,G). We stained the lung tissue sections with anti-ELA2 (human neutrophil elastase) antibody to confirm that the infiltrated cells were mainly neutrophils. The IHC and immunofluorescence results confirmed the observation from H&E staining (Figures 1B,D). We also used the methenamine silver staining (MST) and Calcofluor white staining (CWS) to confirm fungal infection (Figures 1C,D). Interestingly, although there was significantly higher infiltration of neutrophils in the lung interstitial tissue from patients with fungal infections than controls, the blood test showed that the neutrophil numbers and percentage in the blood remained within the normal range (Figures 1E,F). These results indicated that the fungal infection caused more local neutrophilic reactions than systemic ones.

### Germination of *Aspergillus fumigatus* Conidia Into Hyphae in Human Umbilical Vein Endothelial Cells

**FIGURE 2:**
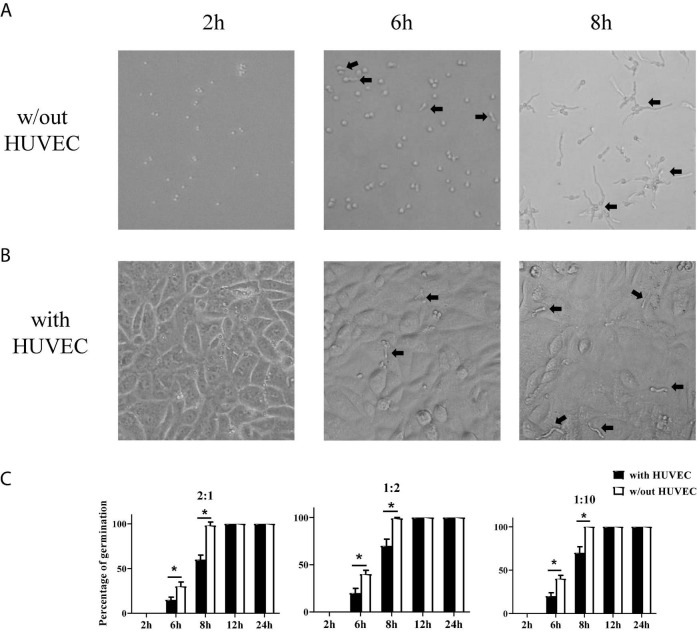
Germination assay of conidia of *A. fumigatus* co-cultured with human umbilical vein endothelial cells (HUVECs). HUVECs were seeded in a 24-well plate (3 × 10^5^ cells per well) overnight before the conidia of *A. fumigatus* were added to the wells at the indicated cell:spore ratio. Representative pictures of conidia at a 1:2 ratio taken at the indicated time with **(A)** or without **(B)** HUVECs. **(C)** The percentages of germinated conidia with added cell:spore ratio at indicated time points were calculated. *p < 0.05. Original magnification × 100. Black arrow: germinating conidia.

Stromal cells are the first line of defense against invading pathogens in the mucosal tissue. *A. fumigatus* infection-related lung diseases are usually caused by the colonization of inhaled conidia of *A. fumigatus* in the lower airways and germinate into hyphae which became invasive. Invasive aspergillosis is usually accompanied by fungal invasion of blood vessels. To investigate how stromal cells respond to conidia stimulation, we added conidia of *A. fumigatus* to HUVECs to test whether stromal cells themselves could limit the germination of *A. fumigatus* conidia. We trialed various doses of conidia (cell:spore ratios of 2:1, 1:2, and 1:10), added to HUVECs, and monitored the germination at different time points (2, 6, 8, 12, and 24 h). The results showed that HUVECs only slightly reduced the germination of conidia up to 8 h. From 12 h onward, all conidia germinated with or without the presence of HUVECs (Figure 2). In addition, the cell:spore ratio of 1:2 was selected for further experiments as at this ratio, we observed 90% of germinated conidia (Figure 2C).

### Human Umbilical Vein Endothelial Cells Upregulate Colony-Stimulating Factor 3 Gene Expression in Response to *Aspergillus fumigatus*

**FIGURE 3:**
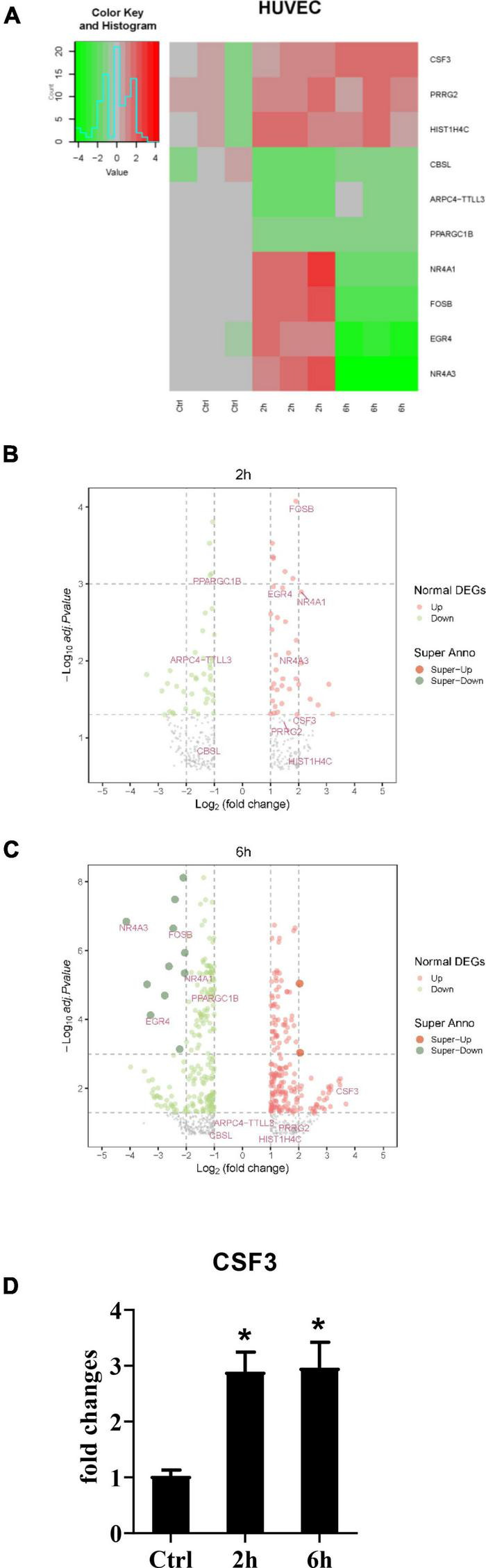
RNA-seq data of HUVECs after stimulation with conidia of *A. fumigatus*. HUVECs were seeded in a six-well plate (2 × 10^6^ cells per well) overnight before the conidia of *A. fumigatus* were added to the wells at a cell:spore ratio of 1:2. The cell lysates were harvested 2 and 6 h post-stimulation and sent for RNA sequencing. The heat-map **(A)** and volcano plots of 2 h **(B)** and 6 h **(C)** are shown. **(D)** The RNA expression of colony-stimulating factor 3 (*CSF3*) was analyzed by quantitative polymerase chain reaction (qPCR). *p < 0.05.

After establishing that the HUVEC cells alone could not stop the germination of *A. fumigatus* conidia, and considering the fact that most people inhaled hundreds of spores each day without any symptoms, it must be the immune response *A. fumigatus* conidia triggered that protected most people from pulmonary aspergilloma. To investigate how stromal cells initiate the downstream immune responses, we stimulated the HUVECs with conidia from *A. fumigatus*, harvested the cells at 2 and 6 h time points, and sent them for RNA sequencing. The RNA-seq data showed that *CSF3* is among the upregulated genes (Figures 3A–C). We also performed qPCR to confirm this finding (Figure 3D). Interestingly, our first observation from patient samples revealed that neutrophils are the most abundant immune cells in and around the *A. fumigatus* infection foci (Figure 1G), and G-CSF, which *CSF3* encoded, is a key cytokine in the regulation of the differentiation, migration, and functions of neutrophils.

### Colony-Stimulating Factor 3 Is Not Required in the Limitation of *Aspergillus fumigatus* Hyphae *via* Human Umbilical Vein Endothelial Cells

**FIGURE 4:**
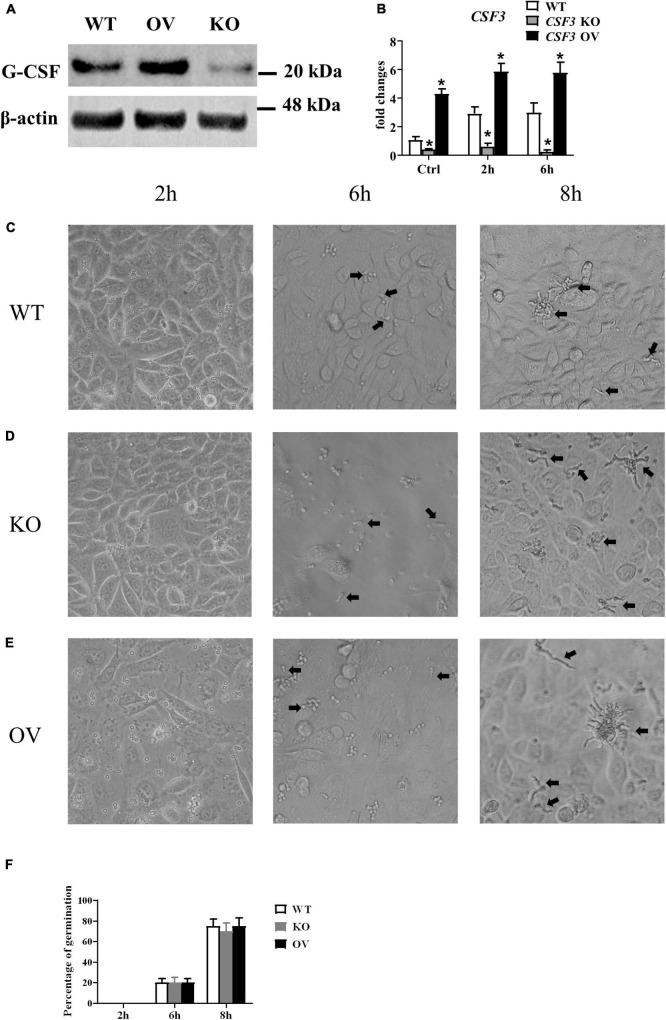
Germination assay of conidia of *A. fumigatus* co-cultured with HUVECs with different expressions of *CSF3*. HUVECs were seeded in a 24-well plate (3 × 10^5^ cells per well) overnight before the conidia of *A. fumigatus* were added to the wells at a cell:spore ratio of 1:2. The protein level of granulocyte-colony-stimulating factor (G-CSF) in HUVECs was measured by Western blotting **(A)**, and the mRNA expression of *CSF3* were measured by qPCR **(B)**. Representative pictures of spores taken at indicated times with WT **(C)**, *CSF3^– /–^* **(D)** or *CSF3*^overexpressing^** **(E)** HUVECs. The percentage of germinated spores was then calculated **(F)**. WT, wild type HUVEC; KO, *CSF3* knockout HUVEC (described in Supplementary Figure 2); OV, HUVECs transfected with *CSF3* plasmid 48 h before stimulation with conidia. *p < 0.05 compared with WT. Original magnification × 100. Black arrow, germinating conidia.

To investigate the role of *CSF3* in *A. fumigatus* infection, we used siRNA to knockdown *CSF3* expression in HUVECs and repeated the conidia germination assay on these cells. The results showed that knockdown of *CSF3* in HUVECs did not affect the germination of conidia (Supplementary Figures 1A,D). Next, we used the CRISPR/Cas9 technique to generate two *CSF3* knockout HUVEC cell lines (Supplementary Figures 2, 4). We also used a plasmid to induce the *CSF3* overexpression in HUVECs. The germination assay performed on these cells showed that *CSF3* knockout or overexpression also did not affect the ability of HUVECs to prevent the germination of *A. fumigatus* conidia (Figure 4). The results showed that *CSF3* upregulation in HUVECs after *A. fumigatus* stimulation does not contribute to the endothelial cell restriction of *A. fumigatus* conidial germination.

### Granulocyte-Colony-Stimulating Factor Released by Human Umbilical Vein Endothelial Cells Affects Neutrophils Functions

**FIGURE 5:**
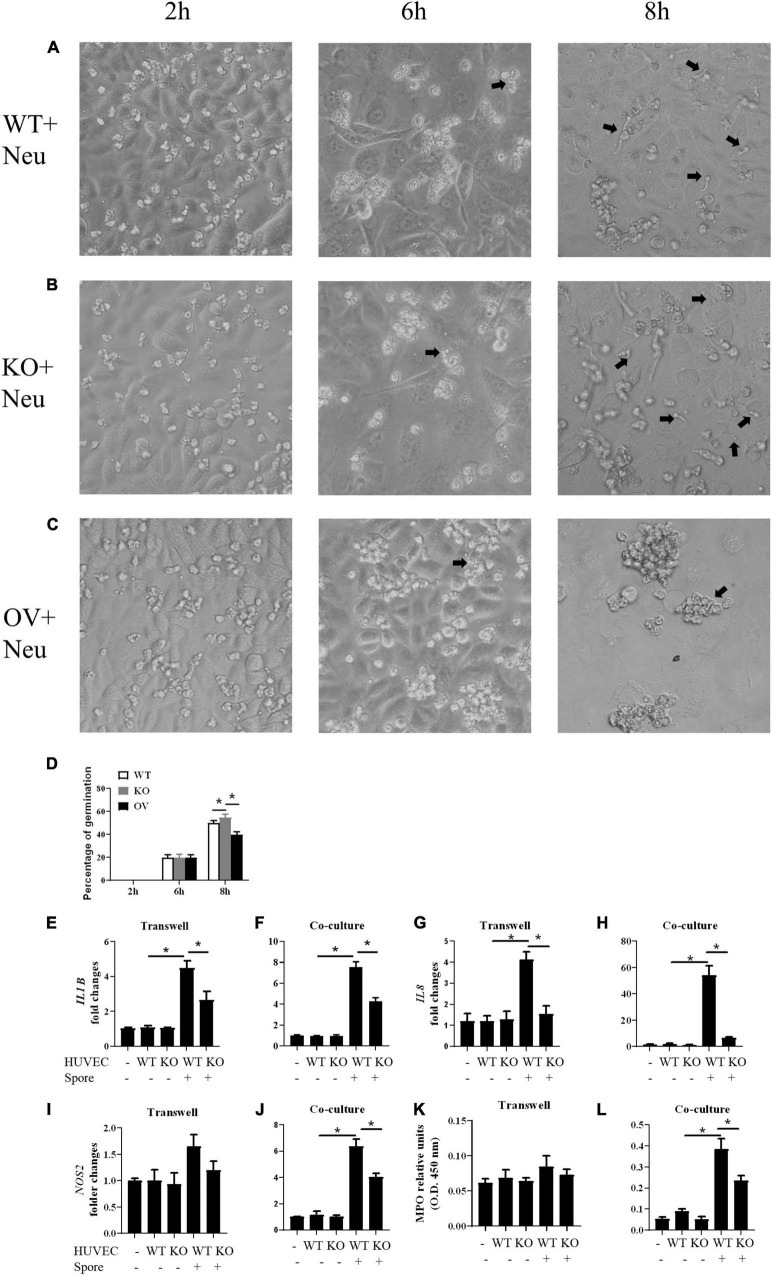
Germination assay of conidia of *A. fumigatus* co-cultured with HUVECs and neutrophils. HUVECs were seeded in 24-well plate (3 × 10^5^ cells per well) overnight before the conidia of *A. fumigatus* were added to the wells at a cell:spore ratio of 1:2. Neutrophils (2 × 10^5^ cells per well) were added 2 h after adding conidia. Representative pictures of conidia were taken at indicated time with WT **(A)**, *CSF3^– /–^* **(B)** or *CSF3*^overexpressing^** **(C)** HUVECs and neutrophils. The percentage of germinated conidia was calculated **(D)**. **(E–L)** Neutrophils (2 × 10^5^ cells per well) were added to a 0.4-μm Transwell chamber **(E,G,I,K)** or without the Transwell chamber **(F,H,J,L)** 2 h after adding conidia, and both the neutrophil cells and supernatant were harvested 6 h later. The gene expression of *IL1B* **(E,F)**, *IL8* **(G,H)**, and *NOS2* **(I,J)** were tested by qPCR, and granulocyte-colony-stimulating factor (MPO) was also analyzed **(K,L)**. WT, wild type HUVEC; KO, *CSF3* knockout HUVEC (described in Supplementary Figure 2); OV, HUVECs transfected with *CSF3* plasmid 48 h before stimulation with conidia. *p < 0.05 compared with WT. Original magnification × 100. Black arrow, germinating conidia.

We investigated whether the G-CSF released by HUVECs could affect the abilities of neutrophils. We stimulated the *CSF3^–/–^*, WT and *CSF3*^overerxpress^** HUVEC cells with conidia of *A. fumigatus*, and the neutrophils were added to the culture. The results showed that neutrophils could prevent most of the conidia from germinating in the presence of G-CSF. Still, the ability of neutrophils was dramatically decreased when co-cultured with *CSF3^–/^*^–^ HUVECs, while it was enhanced by *CSF3*^overerxpress^** HUVEC cells (Figures 5A–D and Supplementary Figures 5C,D).

Next, we used qPCR to analyze the gene expression of *IL1B*, *IL8*, and *NOS2* in neutrophils. To show that the gene expression changes depend on the contacts between the conidia and neutrophils, we used a Transwell chamber to isolate the neutrophils from the conidia. A comparison between the gene expression of neutrophils with (co-culture) or without (Transwell) contact with conidia of *A. fumigatus* showed that even in the presence of stimuli, neutrophils still need G-CSF to upregulate inflammatory genes such as *IL1B*, *IL8*, and *NOS2* (Figures 5E–J). The MPO assay also showed a similar trend (Figures 5K,L). These results indicate that *CSF3* is closely correlated to neutrophil activation.

### Granulocyte-Colony-Stimulating Factor Is Involved in Interactions Between Neutrophil and *Aspergillus fumigatus*

**FIGURE 6:**
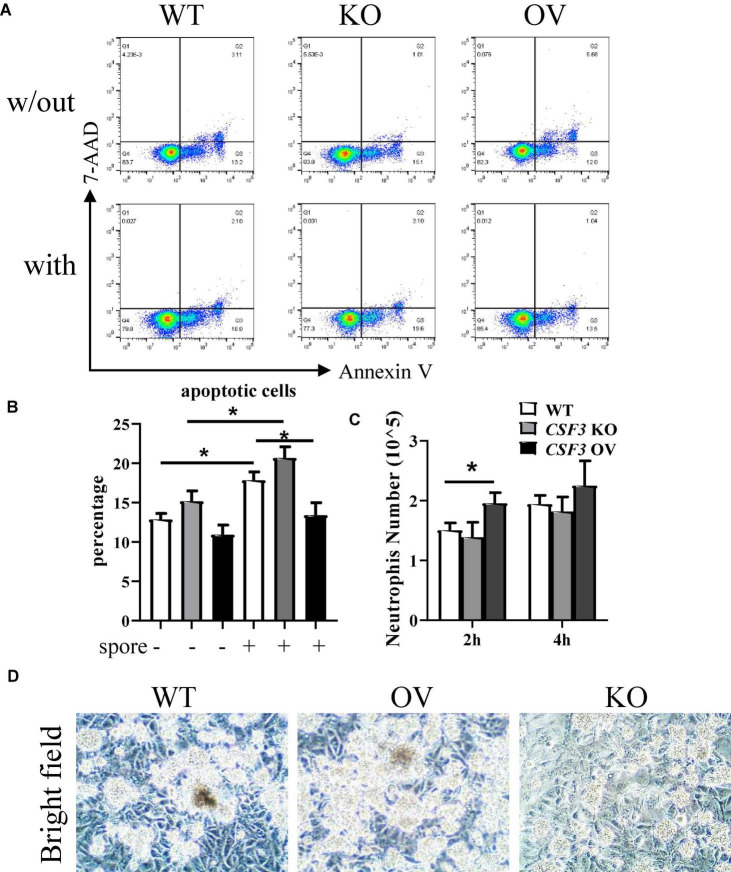
G-CSF has effects in neutrophil functions. HUVECs were seeded in a 24-well plate (3 × 10^5^ cells per well) overnight before the conidia of *A. fumigatus* or control saline were added to the wells at a cell:spore ratio of 1:2. Neutrophils (2 × 10^5^ cells per well) were added to a 0.4-μm Transwell chamber **(A,B)** or a 3-μm Transwell chamber **(C)** 2 h after adding conidia. Neutrophils were analyzed by flow cytometry for apoptosis, representative dot plots are shown **(A)**, and the percentage was calculated **(B)**. Neutrophils that migrated into the lower layer of the well were also quantified **(C)**. Representative pictures of conidia, HUVECs, and neutrophils obtained by light optical microscopy are also shown **(D)**. w/out, without the conidia; with, conidia were added; WT, wild-type HUVEC; KO, *CSF3* knockout HUVEC (described in Supplementary Figure 2); OV, HUVECs transfected with *CSF3* plasmid 48 h before stimulation with conidia. Original magnification × 100 **(D)**. *p < 0.05.

To investigate the interactions between neutrophils, HUVECs, and *A. fumigatus*, we used flow cytometry to analyze the apoptosis of neutrophils after stimulation with *A. fumigatus* conidia. More neutrophils became apoptotic in response to conidia, and this was further enhanced when co-cultured with *CSF3^–^^–^* HUVECs but reduced by *CSF3*^overerxpress^** cells (Figures 6A,B and Supplementary Figures 5A,B). Neutrophil migration was also enhanced by G-CSF released by HUVECs (Figure 6C).

One of the critical methods used by neutrophils to eliminate pathogens is releasing of NETs. As NETs released by neutrophils mainly contains DNA, histone proteins, fiber, etc., we analyzed the extracellular DNA *via* staining of propidium iodide (PI). As shown in Supplementary Figure 3, there were more extracellular DNAs in the *CSF3* overexpression group and fewer extracellular DNAs in the *CSF3* knockout group. In addition, the neutrophils seemed to be less clustered in co-culture with *CSF3^–/–^* HUVECs than WT HUVECs (Figure 6D). These results indicate that *CSF3* is one of the crucial cytokines in regulating neutrophil function during anti-fungal immune responses.

## Discussion

In this work, we showed that HUVECs produce G-CSF in response to *A. fumigatus* conidia stimulation. In addition, G-CSF produced by HUVECs is required for neutrophils to perform their function to prevent the conidia from germinating into invasive hyphae. In response to conidia and G-CSF stimulation, neutrophils can directly kill conidia by phagocytosis and releasing NETs, while producing inflammatory cytokines to recruit and activate other immune cells.

Aspergillosis, which is caused mainly by *A. fumigatus*, is still associated with unacceptable mortality rate despite recent advances in medical research and the availability of new anti-fungal medication (Bandres et al., 2021). This might be due to co-infection with other pathogens such as *P. aeruginosa*, *S. aureus*, *S. pneumoniae*, etc., which were not unusual in immunocompromised patients. These co-infected pathogens might release cytotoxic substances that cause damages to the epithelium, favoring the colonization and germination of *A. fumigatus*. The medicine used for other co-infected pathogens might also counteract anti-fungal medicine (Lv et al., 2021). In this study, we found that *A. fumigatus* could stimulate G-CSF production, which contributed to the activation of neutrophils with lower apoptosis rate (Figures 6A,B and Supplementary Figures 1B,C), which implies a pleiotropic role of G-CSF and neutrophils during infections.

Neutrophils are the first leukocyte type to arrive upon infection, and their proliferation, migration, and functions are tightly regulated by G-CSF (Martin et al., 2021). Neutrophils might be the most important immune cells in terms of the control of *A. fumigatus* infections in healthy subjects (Lehman and Segal, 2020). A healthy immune system has many negative regulatory mechanisms to constrain the activation and life-span of neutrophils (Azcutia et al., 2017; Silvestre-Roig et al., 2019). This is because the ROS, NETs, and other substance neutrophils released to eradicate pathogens could also cause damage to self-tissue (Mantovani et al., 2011; Papayannopoulos, 2018). In immunocompromised patients, the immune system also loses some of its abilities to self-regulate, and the dysfunctional immune responses and prolonged infections combined together may be the reason why they are actually more prone to autoimmune related diseases (Arason et al., 2010; Roe, 2021). This cannot be overlooked during the treatment of immunocompromised patients. This work showed enhanced neutrophil migration and functions by the G-CSF released by HUVECs, which indicated that local instead of systematically administration of G-CSF might eliminate invading *A. fumigatus* with limited side effects, at least during the early stages of infection.

Neutrophils account for approximately 60% of nucleated cells in the bloodstream, and they play a pivotal role in immune responses against invading pathogens, such as bacteria and fungi. Patients with neutrophil disorders always suffer from recurrent or severe bacterial or fungal infections (Martin et al., 2021). One common example is pulmonary aspergillosis, which can only be seen in patients with immune system dysfunctions, such as patients with neutropenia and hematopoietic stem cell transplantation (HSCT) recipients. Neutrophils use several tools to combat invading fungi, such as phagocytosis *via* the activation of FcγR, CR3, or PRRs; the release of reactive oxidants and non-oxidants, and generation of NETs with web-like structures (Lehman and Segal, 2020). However, over-activated neutrophils can cause some non-specific damage to self-tissues, as observed in gout (Vedder et al., 2020), asthma (Ray and Kolls, 2017), pustular psoriasis (Marzano et al., 2019), and rheumatoid arthritis (Thieblemont et al., 2016); neutrophils also could mediate hyper-inflammation accompanied by infections, including SARS-CoV-2 (Cavalcante-Silva et al., 2021). Thus, the activation of neutrophils must be well calibrated and terminated quickly once the pathogens have been eradicated (Lehman and Segal, 2020). In the present study, our results showed that the conidia from *A. fumigatus* could stimulate stromal cells to secrete cytokines to activate neutrophils, leading to tissue damage after long-term exposure to fungi.

G-CSF, a ∼20-kDa glycoprotein encoded by the *CSF3* gene, is the most essential cytokine in the development of neutrophils. In the bone marrow, granulocyte–macrophage progenitor cells require G-CSF to differentiate into neutrophils (Mehta and Corey, 2021). Recombinant human G-CSF has been used to treat severe congenital neutropenia (Skokowa et al., 2017), chemotherapy-induced neutropenia (Mehta et al., 2015), and in HSCT recipients as well. However, this treatment has mixed outcomes (Gupta et al., 2021; Mouchemore and Anderson, 2021). In this study, we found that the stromal cells could release G-CSF upon stimulation with *A. fumigatus*. The increased neutrophil numbers were only seen locally rather than systemically in patients with pulmonary *A. fumigatus* infections. We also observed that G-CSF reduced neutrophil apoptosis. These findings are particularly interesting since *CSF3* is reported to be selectively highly expressed in the lung (Amemiya et al., 2019).

Nevertheless, the roles of G-CSF and neutrophils during infection are very complicated. G-CSF is reported to be beneficial in a mouse model of *A. fumigatus* airway infection (Polak-Wyss, 1991; Ralph et al., 2021). However, G-CSF might not simply associate with similar protective effects in humans. Although neutrophil killing of *A. fumigatus* and *R. arrhizus*, but not *C. albicans*, was enhanced by G-CSF for neutrophils from healthy donors (Roilides et al., 1993; Liles et al., 1997) *in vitro*, neutrophil swarming was rescued for neutrophils from immunocompromised patients (Barros et al., 2021) or from healthy donors but during ROS or MPO inhibition (Hopke et al., 2020), which promoted the usage of G-CSF for immunocompromised patient against opportunistic fungi. Other studies indicated that G-CSF-activated neutrophils might contribute to the allergic reactions in aspergillosis (Patel and Greenberger, 2019). In neutropenic patients, granulocyte transfusion after G-CSF treatment exhibited no beneficial effect against infections (Price et al., 2015). G-CSF treatment provides no significant improvements in cases of pneumonia (Cheng et al., 2007) or sepsis (Bo et al., 2011). In recent SARS-Cov-2-induced COVID-19, treatment with G-CSF is not recommended even though COVID-19 is associated with neutropenia because of G-CSF-induced inflammation outcomes (Lazarus and Gale, 2021). G-CSF treatment for tumor patients with chemotherapy-associated neutropenia raises more questions as well, as new evidence suggests that G-CSF might exacerbate the immune suppression environment in tumors (Mouchemore and Anderson, 2021). These findings indicate that systematic administration of G-CSF might cause detrimental effects than beneficial effects, especially in terms of prolonged exposure.

In summary, this study revealed that the G-CSF and neutrophil activation did have a role in preventing the germination of conidia of *A. fumigatus* to hyphae. However, the inflammatory cytokines produced by neutrophils and the reduced apoptosis rate of neutrophils themselves raised more questions that require further investigation. We believe that, a detailed understanding of the role of G-CSF and neutrophils in fungal infections may help develop more treatment strategies against fungal infection induced diseases.

## Data Availability Statement

The datasets generated for this study can be found in the GEO repository (GSE191240) (https://www.ncbi.nlm.nih.gov/geo/query/acc.cgi?acc=GSE191240).

## Ethics Statement

The studies involving human participants were reviewed and approved by the Ethics Committee on Human Research of the First Hospital of Jilin University. Written informed consent for participation was not required for this study in accordance with the national legislation and the institutional requirements.

## Author Contributions

LW and DL contributed to the experimental design, securing of funds, and manuscript preparation. LW, DH, and DL supervised the study and critically reviewed the manuscript. WZ and DL contributed to the manuscript preparation. WZ, YW, SS, and DL contributed to the experiments and data analysis. All authors contributed to the article and approved the submitted version.

## Conflict of Interest

The authors declare that the research was conducted in the absence of any commercial or financial relationships that could be construed as a potential conflict of interest.

## Publisher’s Note

All claims expressed in this article are solely those of the authors and do not necessarily represent those of their affiliated organizations, or those of the publisher, the editors and the reviewers. Any product that may be evaluated in this article, or claim that may be made by its manufacturer, is not guaranteed or endorsed by the publisher.
