# Supplementary material for: Suppression of Aspergillus fumigatus Germination by Neutrophils Is Enhanced by Endothelial-Derived CSF3 Production
Source: Front Microbiol. 2022 Apr 29;13:837776. doi: 10.3389/fmicb.2022.837776 (PMC9100814; doi:10.3389/fmicb.2022.837776)
Supplement: Supplementary file 1 [file Data_Sheet_1.PDF]

Supplementary Material

CRISPR/Cas9 mediated knockout of *CSF3* in HUVECs

The brief protocol for CRISPR/Cas9 mediated knockout is 1) Generate a plasmid containing the sequences of gRNA, Cas9, and puromycin resistance genes. 2) Generation of lentivirus containing these sequences by co-transfection of HEK cells and harvesting the lentivirus in the supernatant. 3) Using puromycin to eliminate all un-transfected HUVECs 72 h post-transfection with lentivirus. 4) Dilute the HUVEC cells to a concentration of 20000/ml, and add 100ul/well of dilutions to the first line of the 96-well-plate, then do a 4-time titration of cells into the following line (wells in lines 5, 6, 7, or 8 might only have one cell added). 5) Cell culture was continued for approximately 3 weeks, while the cells were monitored every other day after day 5 to mark the wells that had a single colony of cells. 6) Split the cells in marked wells while they reach more than 80% confluence and use half of the cells for sequencing. 7) Expansion of KO clones with CSF3 mutations based on sequencing results. (Figure S2 and S4)

Figure S1. *CSF3* knockdown has effects in neutrophils functions.

The protein level of G-CSF in HUVECs were measured by western blotting (A). HUVEC cells were seeded in 24-well-plate ($3x10^5$ cells per well) overnight before the conidia of *A. fumigatus* or control saline were added to the wells at a cells:spores ratio of 1:2. Control siRNA or siRNA against *CSF3* were added overnight before the conidia stimulation at concentration of 20μM. Neutrophils ($2x10^5$ cells per well) were added in 0.4μm transwell chamber 2 hours after adding conidia. And the neutrophil cells were analyzed by flowcytometry for apoptosis, representative dots plots were shown (B), and the percentage were calculated (C). Representative pictures of conidia were taken at indicated time (D). The percentage of germinated spores while the neutrophils were added without transwell chambers were calculated (E). Original magnification 100x. Black arrow: germinating conidia.

Figure S2. CRISPR/Cas9-mediated genome editing of *CSF3* in HUVEC cells.

A. Guide RNA (gRNA) was designed to target exon 4 of *CSF3* (ENSG00000108342). B. Sequencing results for cells using control gRNA. C. Sequencing results for *CSF3* knockout cells.

Figure S3. Staining of extracellular DNAs.

Representative pictures of Calcofluor white staining (CWS, in blue), extracellular DNAs stained with propidium iodide (PI, in red) and merged images taken by confocal microscopy are shown. WT: wild type HUVEC; KO: *CSF3* knockout HUVEC (described in figure S2); OV: HUVECs transfected with *CSF3* plasmid 48h before stimulation with conidia. Scale bar: 10μm

Figure S4. *CSF3* knockdown of HUVECs.

(A) Additional two pairs of gRNAs used for CRISPR/Cas9-mediated genome editing of *CSF3* in HUVEC cells. (B) Sequence results of the single clone knockout cells.

Figure S5. *CSF3* knockout has effects in neutrophils functions.

WT HUVECs or *CSF3* knockout HUVECs from figure S4 cells were seeded in 24-well-plate (3x10^5 cells per well) overnight before the conidia of *A. fumigatus* or control saline were added to the wells at a cells:spores ratio of 1:2. Neutrophils (2x10^5 cells per well) were added in 0.4μm transwell chamber 2 hours after adding conidia. And the neutrophil cells were analyzed by flowcytometry for apoptosis, representative dots plots were shown (A), and the percentage were calculated (B). Representative pictures of conidia while the neutrophils were added without transwell chambers were taken at indicated time (C) and the percentage of germinated spores were calculated (D). Original magnification 100x. Black arrow: germinating conidia.

Figure S1

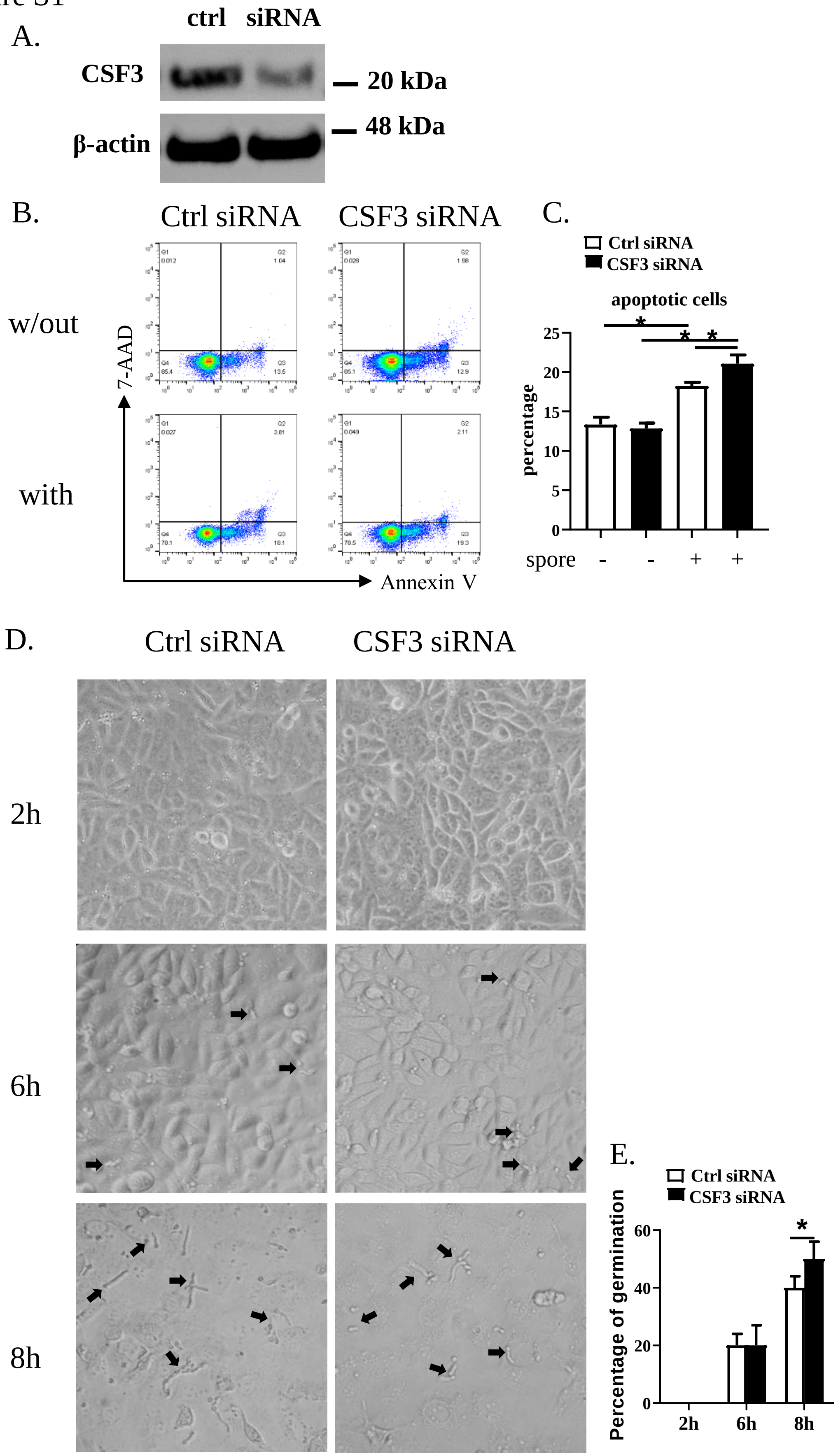

A.
ctrl
siRNA
CSF3
β-actin
20 kDa
48 kDa
B.
Ctrl siRNA
CSF3 siRNA
w/out
with
7-AAD
Annexin V
C.
Ctrl siRNA
CSF3 siRNA
apoptotic cells
percentage
spore
D.
Ctrl siRNA
CSF3 siRNA
2h
6h
8h
E.
Ctrl siRNA
CSF3 siRNA
Percentage of germination
2h
6h
8h

Figure S2

A. Guide RNA designed to target CSF3

Primer ID	Sequence
CSF3	CCGACTTTGCCACCACCATC
Control	CCGGGTCTTCGAGAAGACCT

B. Control HUVECs

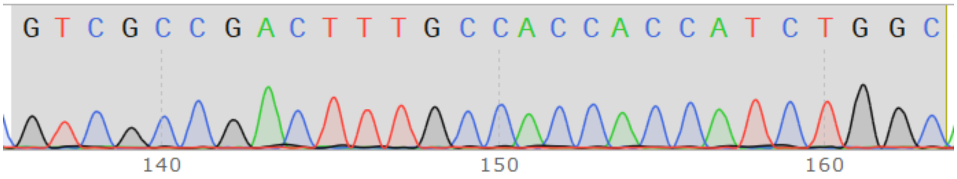

C. *CSF3*$^{-/-}$ HUVECs

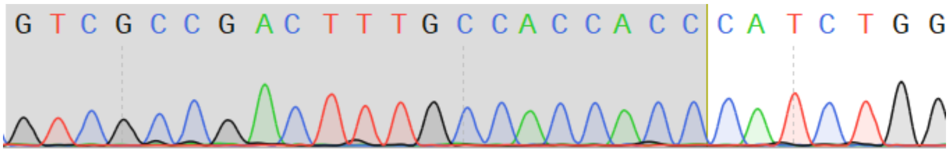

Figure S3

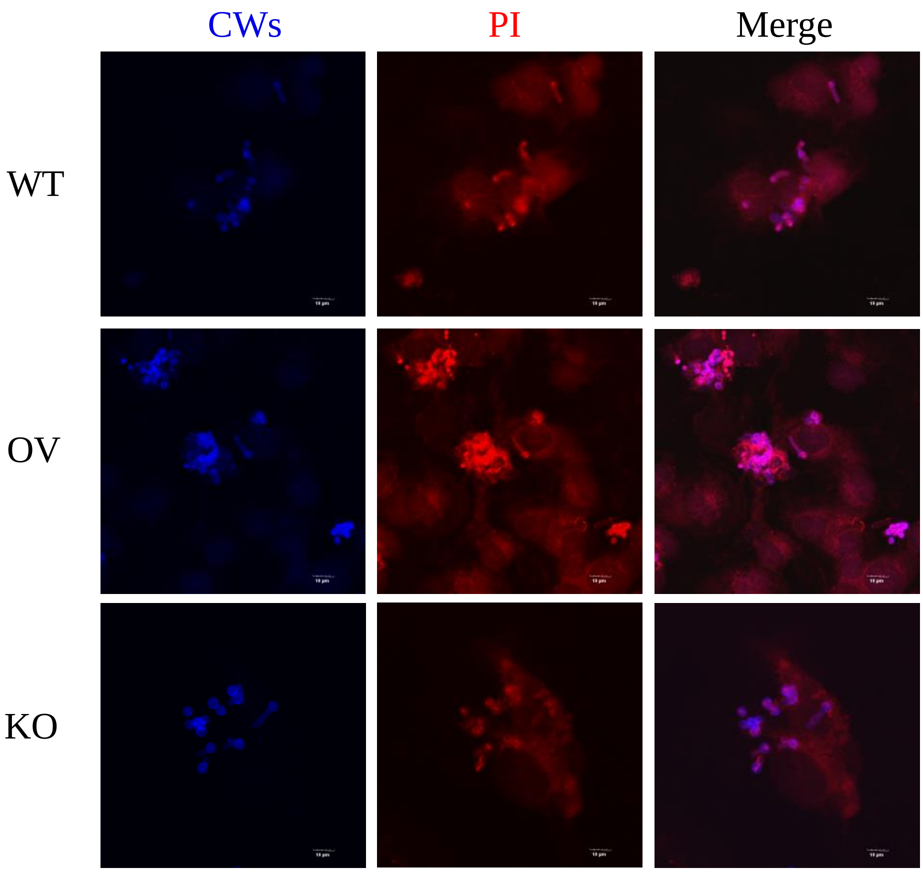

Figure S4

A. Guide RNA designed to target CSF3

Primer ID	Sequence
CSF3_1 REV	aaacCCGAGTTGGGTCCCACCTTGC
CSF3_1 FWD	CACCGCAAGGTGGGACCCAACTCGG
CSF3_3 REV	aaacGGGGGGTGGCTTCCTGCACTC
CSF3_3 FWD	CACCGAGTGCAGGAAGCCACCCCCC

B. Clone 1E4 lost 819bp or 829bp in each alleles

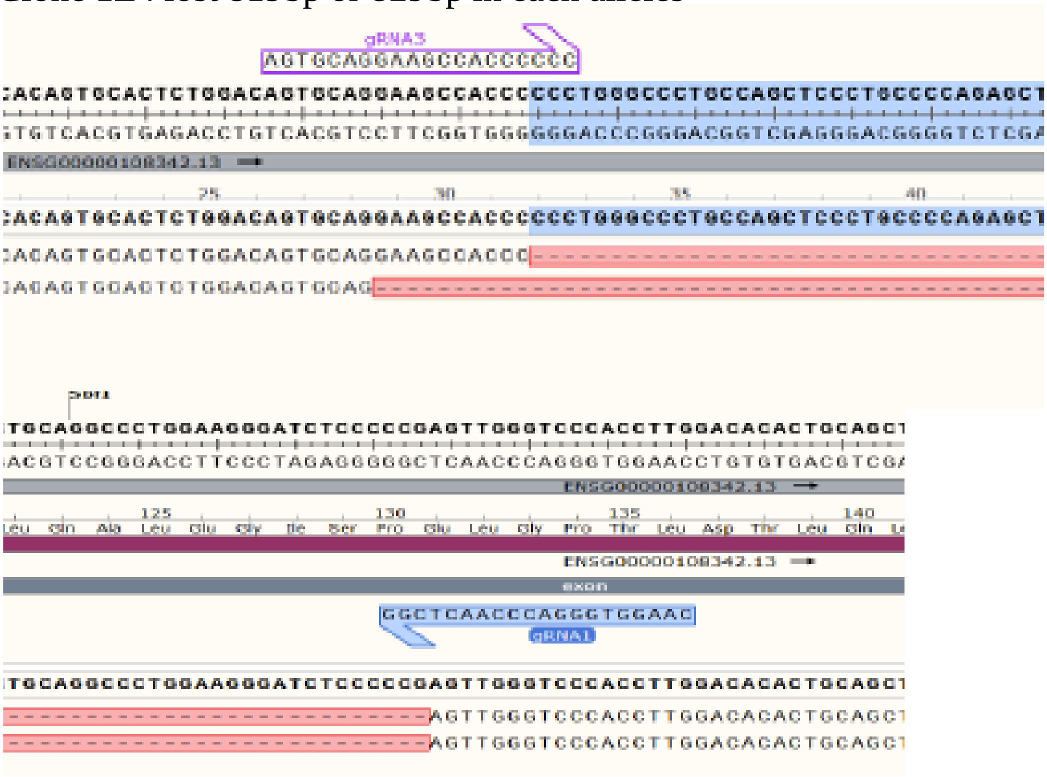

Figure S5

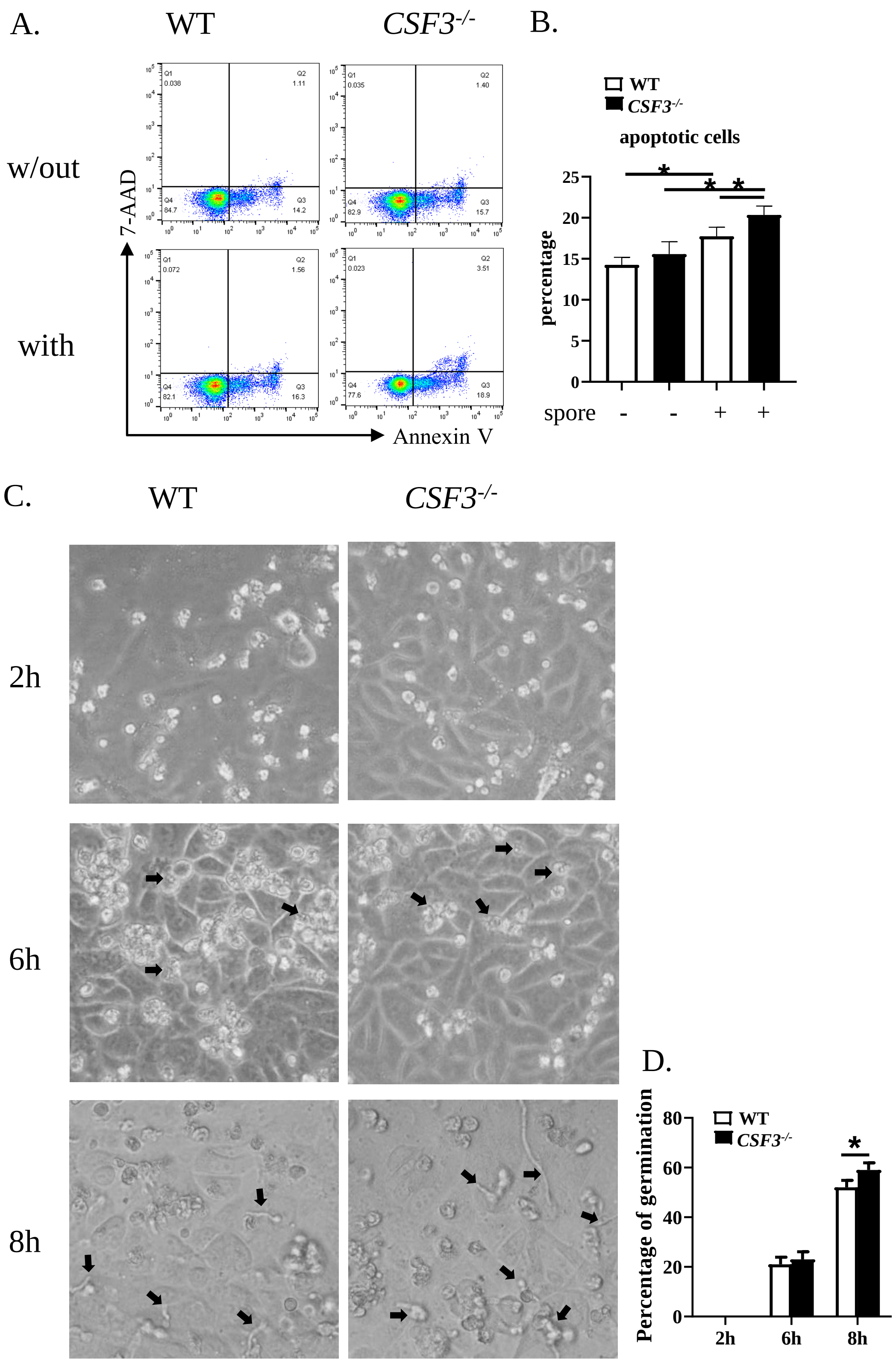

A.
WT
CSF3-/-
w/out
with
7-AAD
Annexin V
Q1 0.038
Q2 1.11
Q4 84.7
Q3 14.2
Q1 0.035
Q2 1.40
Q4 82.9
Q3 15.7
Q1 0.072
Q2 1.56
Q4 82.1
Q3 16.3
Q1 0.023
Q2 3.51
Q4 77.6
Q3 18.9
B.
WT
CSF3-/-
apoptotic cells
percentage
spore
- - + +
C.
WT
CSF3-/-
2h
6h
8h
D.
WT
CSF3-/-
Percentage of germination
2h
6h
8h
